# Beyond the big city: using a systems approach to cultivate a cycling culture in small cities and towns in Ireland

**DOI:** 10.3389/fspor.2023.1127592

**Published:** 2023-06-12

**Authors:** Caitriona Corr, Niamh Murphy, Barry Lambe

**Affiliations:** Centre for Health Behaviour Research, Department of Sport and Exercise Science, South East Technological University, Waterford, Ireland

**Keywords:** systems approach, cycling, active travel, participatory planning, collaborations and partnerships

## Abstract

Urban mobility and how people move in our towns and cities is garnering more attention, as solutions are sought to multiple challenges faced by residents; health and physical inactivity, climate change, air quality, urbanisation and accessibility. Traditional, siloed approaches limit impact and collaborative, systems approaches hold promise. However, systems approaches often remain theoretical and few practical applications of their added value have been demonstrated. This study illustrates how a systems approach can be used to underpin the development of a 9-step process to generate solutions for action on active mobility. The development of a systems map and a theory of change framework are key outputs of this 9-step process. The purpose of this paper is to describe how a systems map was developed in an Irish town utilising broad stakeholder engagement to map the variables that influence cycling in the town and to identify the leverage points for transformational interventions.

## 1. Introduction

Ireland is a car dependent society with one of the lowest levels of active transport and the second highest level of car dependency among EU citizens (1, 2). The national prevalence of active transport has decreased dramatically since the 1986 census when active travel accounted for over 32% of all journeys. Irish census data has indicated that active transport to work and education (primary, secondary and tertiary) has decreased further to a state average of 16.6% in 2016, with public transport use also falling in this time period. Household car ownership has increased its upward trend and in 2016, 81.9% of households across that state owned a car. The National Travel Survey (3),, found that journeys by car accounted for 73.7% of all journeys, rising to over 8 in 10 journeys in thinly populated areas. The most recent Eurobarometer poll (2022), showed Ireland was only one of five EU member states where car dependency levels has increased in the last three years.

In 2021, in Ireland, the transport sector was responsible for 17.7% of Ireland's greenhouse gas emissions, and road transport accounted for 94% of all transport emissions (4). Between 1990 and 2021, transport was responsible for the greatest overall increase in Ireland's greenhouse gas emissions. Transport carbon emissions have become the second largest source of CO_2_ emissions by sector (5). Despite an unprecedented decline in road transport activity as a consequence of COVID 19 lockdown measures in 2020, transport emissions rebounded in 2021, growing by 8%. Global CO_2_ emissions from road transport returned to just 3.6% below the 2019 level in 2021 to 5.86GT of CO_2_ (5). Globally, many cities and urban areas face similar challenges and have unintentionally become car-centric by design, with continued accommodation of car growth through building of additional roads and parking capacity, thereby fostering car use.

Increasingly, cycling is seen as a key part of future transport systems. The global pandemic of COVID 19, the war on Ukraine and subsequent turmoil and uncertainty in energy markets, has increased the urgency of countries managing energy demand through the implementation of behavioural changes related to mobility (6). Both of these phenomena have given rise to new, compelling motivators for cycling. Social distancing and hygiene concerns led initially to a move away from public and shared transports (7, 8). Cycling allowed for avoidance of close contacts and became a valuable alternative to maintain or increase transport capacity (7). These motivators coupled with high fuel costs and uncertain energy supplies have helped to overcome some of the resistance to the reallocation of space for cycling facilities and have enabled rapid deployment of cycling infrastructure in some cities across the world (7).

The co-benefits of a modal shift to cycling are compelling. These include improved air quality, reduced noise emissions, reduced congestion, alternative uses of public space, more equitable mobility options (9) and the well documented health benefits (10, 11). There is an ever growing body of evidence of the impact of cycling on anxiety, stress and vitality (12, 13). As discussed by Patz et al. (14), focusing on the solution to climate change through a health lens may accelerate the shift to a low carbon society and address the greatest health challenge facing developed countries of rising non-communicable disease and decreasing physical activity levels.

The roll out of cycling infrastructure in smaller cities and towns has not been implemented on a similar scale per population to that seen in larger cities worldwide, with the exception of France, where a country wide response saw cycling measures implemented in 207 urban areas (15). The design of smaller urban areas has created a dependency on the private car as the main source of transport, leading to resistance to the reallocation of space necessary for the provision of cycling infrastructure. Smaller cities and towns have fewer resources and smaller teams which can provide greater challenges for implementing sustainable mobility policies. Nonetheless, smaller cities and towns often have well-connected social communities and more walkable and bikeable journeys (16). For smaller cities and towns, cycling presents opportunities to fill the gap in weaker public transport systems, offering park and ride and first and last mile solutions when combined with public transport. The growth in use of E-bikes has also demonstrated the potential to replace longer and more arduous journeys (17). It has the potential to be a public transport offering in its own right and a tourism offering for smaller cities (18).

This action research addressed the challenges faced by smaller cities and towns to reducing car dependency and creating a modal shift to cycling through a systems approach. According to Rutter (19) “systems thinking provides a framework to help examine the factors involved in a problem, the relations between these factors and changes over time”. It has been widely applied to problems arising in complex social, ecological and more recently public health systems. Whilst considering the approach, numerous theories and models, rooted in transport and health and previously applied to examine active travel behaviour were reviewed (20–23). The Physical Activity through Sustainable Transport Approaches (PASTA) consortium reviewed 26 published frameworks and combined behavioural concepts, structural features and a large number of determinants in a single framework. The study concluded that large research projects may still merit a study specific framework (24). Similarly, studies involving policy development have called for study specific frameworks to allow for adaptation to local contexts, the local socio-ecological system, innovation and learning (25). Hence, this action research adopted a systems approach with broad stakeholder engagement to encourage vision-orientated and community-based decision making towards a framework specific to the context.

A systems approach allows for dialogue between stakeholders, enabling them to learn how to change their patterns of decision making and emphasise project-based experimentation and social learning (26). Furthermore, the involvement of stakeholders results in better knowledge, better decisions, capacity building, enhanced social capital and therefore better success (27). The process of enabling idea formation, solution generation and learning, contributes to the sense of ownership and the building of a local platform necessary for implementation. Taken originally from the field of systems science or systems dynamics, this approach has many methods of application and terms are often used interchangeably. The development of a systems map is one common method associated with systems science. The process of drawing the map is as important as the map itself, as stakeholders communicate and collaborate to identify different elements of the system and their interconnections. This broad cross sector coordination can lead to large scale impacts in systems (28). Kohl et al. (29) recommends a systems approach focusing on populations, rather than individuals to increase physical activity worldwide. More recently, an OECD report on Redesigning Ireland's Transport for Net Zero (30), stated that “patterns of behaviour, including mobility, are the product of the systems they are embedded in. Transport systems have been shaped by existing policies but similarly, a shift in policy has the potential to redesign systems and enable large-scale behavioural change.” Similarly, the Intergovernmental Panel on Climate Change (IPCC) seeks transformative change to reverse current patterns of behaviour, “system-wide change that requires more than technological change through consideration of social and economic factors that, with technology, can bring about rapid change at scale” (31).

Following these recommendations, this research developed a pragmatic 9-step process, underpinned by systems science, to develop a cycling culture in Kilkenny city, Ireland. Specifically, the purpose of this paper is to describe the first five steps in the process. This involved multiple methods of stakeholder engagement to develop a systems map of the variables that influence cycling in smaller cities and towns and to identify the leverage points for transformational interventions.

### 1.1. Study area and context

Kilkenny county (population 103,685) is located in the south east of Ireland and it's main urban centre is Kilkenny city with a population of 26,512 (32). It is the 4th largest urban area in the Southern Region of Ireland and has experienced a population growth of 8.6% over the 5 year intercensal period since 2011. The National Planning Framework 2040 envisages a 30% population growth in Kilkenny up to the period of 2040. The population of the county living in urban areas is increasing, from 35.3% in 2006 to 37% in 2011 and to 40% in 2016. However, Kilkenny is still a predominantly rural county in terms of population. The city is the 8th largest employment centre in the state, and is a self-sustaining regional driver, with a daytime working population of 13,738. Kilkenny has excellent road links to Waterford and Dublin. The existing rail links to these cities have limited schedules for commuters, with no service for the morning commuter on the Waterford route. The National Transport Authority introduced a Kilkenny City Bus service late in 2019, with two routes, which has grown to over 5,000 passenger journeys weekly. The city is also served by private bus providers and a demand response service, the Local Link. The projected population growth has the potential to result in a significant increase in the demand for travel in the city with the current modal share (Table 1) (32).

**Table 1:** Modal share targets taken from the kilkenny city and county Development plan (33).

	Walk	Cycle	Public transport	Car
2016 Census	23.3%	3.1%	4.0%	63%
2040 Target	35%	10%	15%	40%

Kilkenny city is predominantly flat and compact with moderate levels of traffic congestion and a population density of 2,115.9 per km^2^. It is approximately 3 km wide and 4.5 km long with many destinations easily accessible by foot or by bike. The County Development Plan (33) has set out ambitious targets for modal share in Kilkenny city, achievable through the implementation of compact growth and the 10 min city concept. Despite ambitious aims and objectives, Kilkenny city has struggled to reduce car dependency. A mobility management plan was adopted in 2009 and an inter-agency Smarter Travel committee was established. Although some major infrastructural projects were delivered, including two additional river crossings, there was no evidence of increased modal share of active modes (34, 35). In Kilkenny city, over 65% of males use motorised transport and 64.54% of females use the car as their means of transport for journeys to work or school. The percentage of those using cycling as a means of travel in Kilkenny fell from 13.65% in 1996 to 3.12% in 2016 with the greatest decline in secondary school students (13 to 18 years) from over 40% in 1996 to just over 3% (3.24) in 2016. Compared to the national average of 4.43% in 1996, Kilkenny had a high percentage of people cycling as a means of transport at 13.65%. In 2016, cycling as a means of travel in Kilkenny was just above the national average of 2.68%.The allocation of 20% of the transport budget to active modes in 2020 and the installation of active travel teams in the local authorities presents new opportunities to drive transformational change. The researcher is embedded in the local authority, alongside an Active Travel team of three staff. The reach of this team is extended through an inter-departmental steering group and a broad stakeholder group, focusing on sustainable transport in the city.

### 1.2. Methodology

#### 1.2.1. Research design

**Figure 1:**
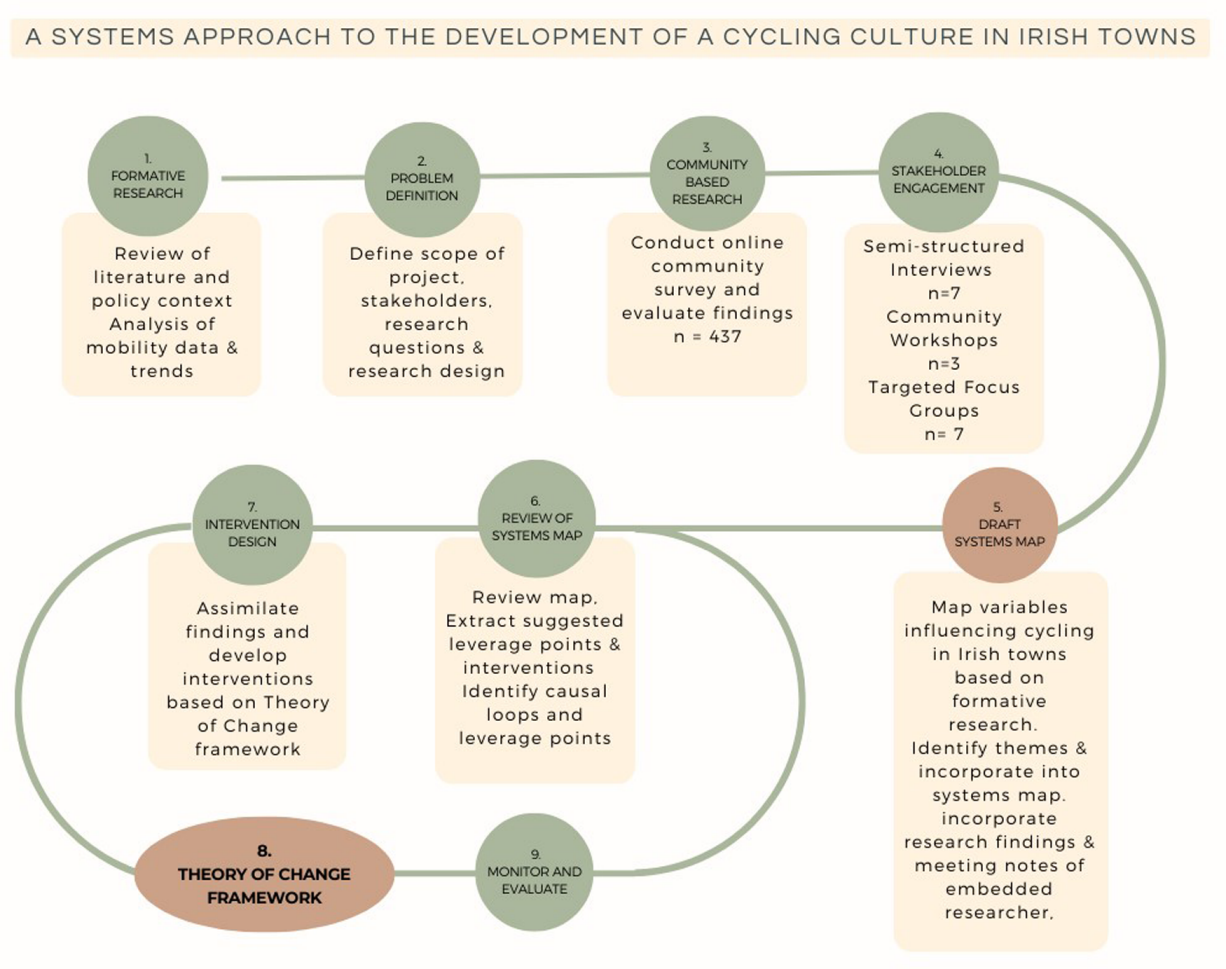
The 9-step process used for developing a systems map and theory of change framework.

The research consisted of creating a systematic 9-step process designed to develop two key outputs; a systems map and a theory of change framework, based on Nutbeam and Bauman's evaluation framework (36). These steps, related activities and how they are aligned are illustrated in Figure 1 below. Step 1, the formative research, was conducted encompassing a review of the literature, policy context and planning framework and an analysis of mobility data and trends. This step allowed the researcher to build on existing research and gain an understanding of the local context, mobility patterns and trends. The detailed step 1 findings are beyond the scope of this paper and are presented in full on the project website (37). This paper describes the development of the first key output, the systems map (steps 2–5). Steps six to nine, relate to the second key output, the theory of change framework, where the systems map is translated into a pragmatic format easily used by stakeholders and familiar to practitioners. Due to the depth of the engagement and length of the process, this will be published separately. This research was approved by the research ethics committee of Waterford Institute of Technology (WIT2021REC014).

## 2. Methods

This section describes the methods, and where appropriate, the data analysis, for steps one to five.

### 2.1. Step 1: formative research

A literature review was conducted to identify the factors that influence cycling in cities and towns. A google scholar alert was set up using the following keywords; utility cycling, cycling for transport and active travel. Frameworks, determinants and factors influencing cycling were reviewed from 2016 onwards. The European, national and local policy and planning frameworks were reviewed and objectives relating to transport planning and active travel were documented.

Mobility trends in the European, Irish and local context were identified through extraction of data from the following sources; the Eurobarometer (1, 2), the National Travel survey, the Irish Sports Monitor dataset (38) and the Census of Ireland (32). Modal share of all means of travel was calculated and comparisons were made by towns of similar size, gender and age groups between 1986 and 2016.

### 2.2. Step 2: problem definition

Research team meetings and meetings with the research partners and other stakeholders allowed for deliberation and definition of the research objectives. Initial discussions were through the Cycle Kilkenny stakeholder group, established by the Local Development Company to explore Cycling in the City. As the process evolved, the discussions were shaped by the development of the Local Transport Plan, incorporating active travel objectives, led by the local authority. The functional area of the research was defined, the stakeholders involved were identified and the research objectives were identified.

### 2.3. Step 3: community based research

The community wide survey was conducted to determine the needs of the community for mobility in the city, liveable streets and public spaces and to identify the barriers and enablers of cycling. The survey contributed to step one and two, the formative research and the problem definition. Furthermore, the survey gathered evidence on public attitudes to develop the narrative on how and why cycle planning will meet a range of public interest and stakeholder objectives. Lastly, the survey created awareness in the community of the project's goal, to cultivate a culture of cycling within the community, and thereby gained community support and involvement and enhanced the collaborative process.

The community wide survey was conducted in June and July 2019. Ireland was re-opening following a full lockdown and moving into Phase 2 of Covid-19 restrictions; people were now allowed to travel within their county and meet up to six people from outside their household. Organised indoor events were not permitted and working from home was to continue where possible. For these reasons, the survey was hosted on the Survey Monkey platform, a non-probability (convenience) sample was generated (*n* = 437). The sampling method was unrestricted and self-selected (39). The survey was piloted (*n* = 30), final edits were made and the survey was circulated through social media platforms of the organisations represented in the original Smarter Travel committee.

Specifically, the survey consisted of 35 questions categorised into six distinct sections. The first section contained questions on participant demographics taken from the Irish census (2016). Additional items such as physical activity (40), bike and car ownership and residential location were also included. Community accessibility was measured using the Perceptions of the Environment in the Neighbourhood Scale (41, 42). Two questions were amended to be more cycling specific and reflect more recent findings suggesting the importance of segregation (43) and network connectivity (44–46). The community attitudes to cycling section was a seven item measure, based on the theory of planned behaviour with the additional items to capture habits and perceived social norms (42, 47). Community needs were assessed with questions adapted from The Town Centre Living Initiative (48) and a Town Centre Assessment carried out under the Intereg programme by South Ayrshire Council (49). The section on barriers and enablers consisted of variables that have been widely reported in the literature and in recent national surveys conducted in two low cycling countries; Scotland and New Zealand (3, 50). Cost of parking at work and trip chain information variables have been included based on previous research in Kilkenny (51). Lastly a question on the propensity for modal shift was adapted from the Switch Tool Kit (52).

### 2.4. Step 4: stakeholder engagement

The stakeholder engagement was designed to elicit a greater understanding of the complex issues and the nuances that shape cycling patterns in a local context. Critically, a rich collection of experiences was gathered and diverse stakeholders were engaged early in the participatory planning of interventions. An in-depth qualitative study was conducted using several methods of data collection to ensure a broad and diverse engagement and to capture the voices that are often unheard. These methods included semi-structured interviews with key stakeholders, community workshops, targeted focus groups and reflective note taking following meetings and events throughout the engagement process. This approach enabled the researcher to understand the challenges faced by key stakeholders working at the intersection of transport and health. Throughout the process, stakeholders were encouraged to share potential solutions to mobility challenges, to identify possible leverage points and suggest interventions.

For participation in the stakeholder engagement, respondents were contacted in advance, by email or phone, and invited to participate. Upon confirmation of attendance, follow up emails were sent to obtain informed consent and permission for recording, to describe the purpose of the study and present the interview or topic guide schedule. Interviews were recorded using a dictaphone or through the zoom platform. All interviews and workshops were transcribed verbatim, de-identified and imported into NVivo (released in March, 2020). Researcher notes and memos were also imported into NVivo.

#### 2.4.1. Semi-structured interviews

Semi-structured, face-to-face interviews were conducted with key stakeholders in May and June of 2020. The set of stakeholders represented those in key decision maker roles at national and regional level, cycling advocates and city centre traders (*n* = 7). An interview guide was developed based on the researcher's knowledge of the factors influencing cycling and the local context. The interview guide prompted respondents to describe their ideal city and possible mobility solutions, to discuss challenges to mobility and the necessary steps to help overcome these challenges. Interviewees were encouraged to share their unique personal and professional experiences of mobility and cycling in the city.

#### 2.4.2. Community workshops

Community workshops were conducted through three channels, the Public Participation Network (PPN); a forum for citizens involvement in decision making, the Chamber of Commerce, and Cycle Kilkenny; a group of organisations, including the local authority, working together to promote cycling in Kilkenny. The workshops moved to an online format on the zoom platform due to COVID-19 restrictions. Invitations were extended and permissions were sought through the three organisations. The workshops all followed a similar format. Firstly, information was presented on the demographic and travel trends, mode shares and the local policy context. Depending on the number of attendees, questions were then posed to all attendees or smaller groups with facilitators, with targeted probing of issues specific to the experiences and expertise of the participants. Issues arising varied greatly with the diverse stakeholders. Existing cyclists focused on the difficulties experienced moving through the city, cycling infrastructure deficiencies and the ideal cycling city. Non-cyclists discussed the barriers they faced to cycling, the forced car-dependency, the lack of alternative mobility choices and possible solutions. Participants were invited to use the chat function throughout the workshops. The smaller workshops were recorded and transcribed. Note-taking was used with break-out rooms and additional facilitators for the larger workshops.

#### 2.4.3. Targeted focus groups

The online nature of the survey led to under representation of certain cohorts of the population. These were identified through a comparison of the respondent's data with the population demographics from the census. Typically, those missing or under-represented were the cohorts of the population that encounter limitations in how they engage with and move through the cityscape and those who traditionally face barriers to engagement. A targeted series of seven focus groups were conducted with the following participants: people with chronic health issues, people with an intellectual disability, people with physical disabilities, older adults, single parent families, members of the travelling community and members of migrant communities. The workshops and conversations focused on needs and experiences in regards to mobility, accessibility and public space. These workshops were conducted in partnership with the enterprise partner and analysed separately. The findings captured the experience of individuals and together the individual perspectives created a holistic picture and understanding of how people move through and interact with their city (53).

#### 2.4.4. Reflective note taking

The stakeholder engagement process was enriched by the embedment of the researcher alongside the newly formed active travel team. This enabled the researcher's involvement in, and attendance at planning and project meetings with city traders, elected representatives, strategic policy committees and transport authorities. A Sustainable Transport group was formed for this process with representatives from the local authorities, planning, transport and environment, and other stakeholders. Following three meetings, an application was made to Interreg Europe for a peer review process. This resulted in a two-day workshop with in-depth discussion with five experts in sustainable transport focusing on the mobility needs in Kilkenny. The Interreg Europe team facilitated the workshop with the peers, the local authority and other key stakeholders. Following this, a decision was made to extend the Sustainable Transport Group to a wider group of stakeholders and the local authority began the process of developing a Sustainable Urban Mobility Plan. Insights and understandings were gained throughout this process by the researcher. Further learnings were gained through attendance at a study visit with the Dutch Cycling Embassy undertaken by the Active Travel team and political representatives, meetings with the National Transport Authority and team meetings with the Active Travel team. Regular meetings and bike rides took place with cycling advocates. Over 50 workshops and meetings took place in the engagement process. Reflective notes were recorded during and after meetings and added to NVivo as memos. Prompts for note taking included discussions on challenges faced by the stakeholders, problem definition, and novel and innovative solutions.

##### 2.4.4.1. Stakeholder engagement data analysis

Several data analysis strategies were considered. The envisioned outcome required identifying, analysing and reporting themes to build the systems map which led the researcher to choose thematic analysis. This approach does not begin with a theory or hypothesis to be tested but instead adopts an inductive approach to data analysis (54). Responses were not grouped in predefined categories but rather salient categories were derived from the data itself. Categories emerged through several rounds of coding. Following transcription, documentation of meeting notes and familiarisation with the data (round 1), three additional rounds of coding were applied to the data. This analysis was applied systematically to all qualitative data. The rigor of the process was enhanced by the use of NVivo software analysis for organising and documenting the data analysis process. This platform allowed for the synthesis of the findings of all the qualitative engagement methods. The method used was based on the step-by-step approach of conducting a thematic analysis presented in a study exploring rigor and trustworthiness of qualitative research (55). This method offers a systematic approach that increases the traceability and verification of analysis. Reflective thoughts were captured throughout in memos. In round two, initial codes were generated by two researchers, coding was compared and a coding framework was developed. In round 3, the relevant coded data was sorted and collated. Round 4 reviewed the coded data to identify themes. Themes were collapsed or separated as needed. Themes and subthemes were vetted by team members. Themes were defined and named and the scope and content of each was described. The final themes with supporting quotes are presented in Table 2 and were incorporated into the systems map.

**Table 2:** Themes and sub themes with supporting quotes from all stakeholder engagement data collection methods.

Theme (with supporting quotes)	Sub Theme
Time of change and transformation, build capacity to adapt, new skills sets	
“I think Kilkenny has a really great opportunity for a regional town to turn the narrative around, that’s a narrative that’s embedded in a lot of wider challenges around the future of our town centres.”	Changing social norms for mobility
	Building safe, segregated cycle ways as a catalyst for change
	Overcoming reluctance to discommode vehicular traffic
“The single greatest challenge is fear, I think people have a fear of change, what we’re looking at is a major cultural shift, it’s not tweaking around the edges anymore, what has to happen for a place like Kilkenny has to be quite radical but it has to be done in a way that’s participative and that people are brought on board.”	Overcoming resistance to change
	Responding to the needs of cyclists
	Retrofitting and reallocating space
	Seizing opportunities in a time of change
“The drive is there to do it so I think if the willingness is there to do it amongst all actors, there’s a great opportunity”	
Working structures and statutory processes, evidence based and impartial decision making	
“I don’t doubt the challenges that’s ahead in trying to bring the focus back on to the smaller towns. That’s effectively what we are. For all our lofty ideas about the city and all that, we are still a small regional town and I think we need to continue to look across at potential partners in other European municipalities”	Addressing a skills deficit
	Building capacity to deliver change
	Desiring and striving for high standards
	Developing relationships with funders
“we had city engineers who wanted to make progress on it, we had planners in the planning section who wanted to make progress on cycle infrastructure. I’m not sure if we had the necessary skill set in the Local Authority to see that from a hard road engineering perspective to a cyclists perspective and again there lies the challenge”	Needing political will
	Negotiating local and national policy
	Negotiating planning and political processes
“I think there needs to be a whole of government approach on something like this. The Dept of Education will not see it as their problem or spend anymore money to buy a bigger site that would allow good parking for the bikes or rooms for lockers – but they just don’t see it. There needs to be a shift in their thinking”	
Collaborations, cross sectoral work, multi-disciplinary teams	
“You can’t impose systems on people or on business who are going to be struggling even more now, that they don’t want, so I think it’s really important that we do that and we take on board the views of everyone who has a stake hold in the town”	Needing a multi-stakeholder approach
	Cycling as a cross-department function
	Thinking beyond cycle lanes ancillary cycling supports
	Understanding others roles and perceptions
“It’s nobody roles, Sports Partnerships do a bit, An Taisce do a bit, the independent cycle training providers do a bit it. It falls under everybody and it falls under nobody specifically”	
	Overcoming lack of continuity in roles
Community Engagement, local knowledge, place based solutions	
“it’s about the likes of a champion in certain schools and we pretty soon identified that there were schools that the principal or some of the teachers were cyclist themselves. Cyclists in the terms of recreation or commuter cyclists and then they just get the whole idea and they want more of it”	Cycling initiatives aimed at targeted groups
	Developing localised solutions
	Engaging the wider community
	Modelling behaviours and champions
“Cycle to work…the target market there should be the 10% that are living within 5 or 6 km of the work and it’s not the 500 people in the workplace, it’s the 20 or 30 that we should capture that can easily transfer, that don’t have chain journeys.”	Partnering with schools and workplaces
	Understanding others roles and perceptions
Future proofing, forward planning, monitoring and evaluation	
“We are the right size and they have designed Kilkenny well with the neighbourhoods and 10-minute city piece”	Needing a cycle network and central cycle access route
	Needing and recognising the importance of participatory planning
“Biggest challenge to me is the fact that we are a medieval city and it is mission impossible to provide space for everything and the solution is the notion of making the Nore the link, that you connect to the Riverside walk from wherever you are and then you’re off road and you can cycle to wherever.”	
	Optimising land use to reduce transport demand
	Providing alternatives to overcome car dependency
	Using demand measures to overcome car dependency
	Utilising research and data
Planetary and human health, healthy communities and town centres	
“if you create wow, well, you've made a friend, you're already winning. So if you do the same with your town, you're winning with tourists and if you can make your residents say wow? That's when they take pride and all sorts of things”	Accommodating and encouraging city centre living
	Being a compact and sustainable city
	Preserving and or developing vibrancy of urban centres, Regenerating spaces, Creating the wow factor
“we have to reimagine what our towns look like and what the spaces are for and that includes the periphery, includes the estates on the edge of the town and how that all interconnects”	Generating connectivity, connecting destinations and recognising the needs of the rural dweller
“It’s a real aperitif before work and a refresher after work, 10-minutes of fresh air and leave work behind and look forward to what you are going to do for the evening.”	Connecting with nature and accessing green spaces
	Facilitating independent mobility for all and developing on-road cycling confidence
“As a cyclist you have far more freedom to cover space in town. You tend to visit much more businesses because you can, don’t have to worry about car parking or how long you’re there. You can get from a city centre feeling, hit of fresh air, pop down along the canal very comfortable in amongst the greenery and go back into town”	
	Recognising and maximising the environmental benefits of cycling
	Generating social interactions and building social capital
	Recognising and maximising the health benefits of cycling
	Consumer choice and paying for decisions and modes
Safe and inclusive design and multi-modal solutions	
“it’s very frustrating when you are pushed up onto a footpath coming to a roundabout and you have to decide do I get off the bike, do I cross the road as a pedestrian with a bicycle, do you stay on the roundabout even though you’ve run out of cycle lane”	Access to bike schemes
	Accommodating all users through inclusive and legible design
	Consistent inclusion of groups with specific needs at all planning stages
“My children started cycling when they were about 9 and when they went out the door, my heart was in my mouth. I started cycling with them obviously but they very quickly shrug you off and said they were fine and your heart is in your mouth when they do go off and you teach them the rules of the road and you just hope that someone doesn’t hit them because if they get hit by a car, they are vulnerable”	Cycling initiatives aimed at targeted groups
	Dependable public transport routes with clear communication of routes
	Integrating with public transport
“wouldn’t it be great if the busses had the capacity to put bikes on the back of them”	

### 2.5. Step 5: draft systems map

**Figure 2:**
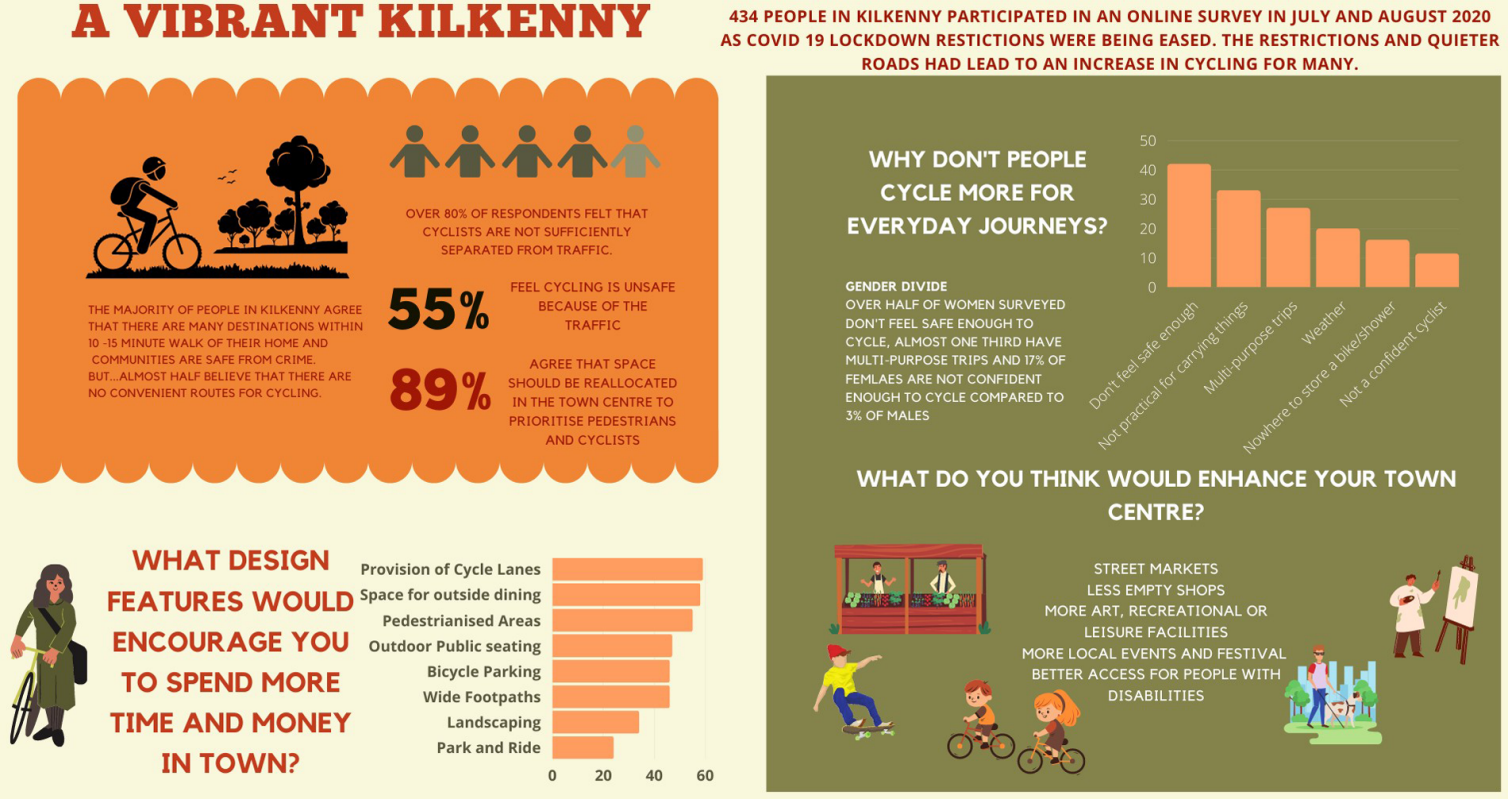
Infographic of survey results.

A systems map was drafted by the researcher based on the findings of the literature review using the KUMU online platform. The initial draft of the systems map was adapted from the Global Action Plan on Physical Activity 2018–2030 (GAPPA). The GAPPA map sets out four strategic objectives; Create Active Societies, Create Active Systems, Create Active People, Create Active Environments. The factors identified from the literature as influencing cycling were added to the maps as elements. The first draft of the map was prepared prior to the stakeholder engagement and was presented to prompt discussion at the community workshops and meetings of the Sustainable Transport Group. The majority of issues arising throughout the community engagement process reflected the findings of the literature. However, following the thematic analysis of the qualitative research, the map was restructured and a fifth strategic objective; Create Safe Environments, was added. Leverage points, places within a complex system where a small shift in one thing can produce big changes in everything (Meadows, 1972), were suggested throughout the engagement and recorded. Building the map was an iterative process, throughout the research, as the researcher worked alongside the Active Travel team and incorporated the learnings from planning and project meetings. The map is presented below in Figure 2.

## 3. Results

This section presents the results of Steps 3, 4 and 5.

### 3.1. Step 1

Findings of the literature review informed the research design and initial draft of the systems map. Mobility trends were summarised and the detailed analysis is presented on the project website together with a review of the policy and planning context (37).

### 3.2. Step 2

Through research team meetings and meetings with the research partners and other stakeholders, the following research objectives were formulated:

• Adopt a systems approach to map the variables that influence cycling in Kilkenny City
• Utilise broad stakeholder engagement to identify the leverage points for transformational interventions
• Develop a theory of change framework to cultivate a cycling culture in Kilkenny

The 9-step process was outlined to achieve these objectives.

The functional area of the research was defined as the two electoral areas in Kilkenny City and the electoral area of Kilkenny rural, covering the functional area of Kilkenny city.

The stakeholders were identified as those already involved in the Cycle Kilkenny stakeholder group; Kilkenny Leader Partnership (local development organisation), Chamber of Commerce, Cycling Advocates, the Local Sports Partnership, Disability Access Group and transport representatives from Kilkenny County Council. Additional stakeholders were added so that the group was representative of the entire population. The stakeholder list was reviewed against the quintuple helix model (56), representing government, academia, industry, civil society and the environment and also against the ‘Topic Guide- Sustainable Urban Mobility Planning in Smaller Cities and Towns. In response to this, an interdisciplinary team was invited from Kilkenny County Council, representing planning, transport, environment, climate action and tourism. Representatives were also invited from large employers, local traders, the Public Participation Network (representatives of community and voluntary groups) and organisations representing young people.

### 3.3. Step 3: community based research

Over 50% of respondents lived in Kilkenny City, a further 17% lived within 5k of Kilkenny, a quarter of all respondents lived between 5 and 20k from Kilkenny and just over 5% live greater than 20k from Kilkenny City. Just over 60% of the respondents were female and 60% of all respondents were between 40 and 60 years of age. 90% of respondents owned a car and 66% had two or more cars. Approximately 12% did not have the use of a bicycle.

Almost 90% felt that their community did not have a problem with crime or anti-social behaviour. The vast majority (88%) of the respondents felt that people in their community can be trusted and almost 70% felt that they knew their neighbours either moderately or extremely well.

When asked about their community and cycling, 55% felt cycling was unsafe because of the traffic and almost half felt that there were no convenient routes for cycling. 82% of all respondents did not agree that cyclists were sufficiently separated from traffic. Five per cent had no interest in cycling, over 40% would like to cycle more and the same number again would like to cycle more if the conditions were right. The most frequently cited barrier was that people don't feel safe enough on the roads. The next three barriers were; not practical for carrying things, journeys are too far and multipurpose trips (transporting children, drop offs etc.).

When asked about their perceptions on cycling, over 90% of all respondents agreed or agreed strongly that people who cycle regularly improve both their health and well-being, that it would be better for the environment if more people cycled and Kilkenny would be a better place if more people cycled. The majority (96%) of all respondents believed that the Local Authority should be investing money in cycling. In spite of the concerns around safety, over half had positive experiences of cycling in Kilkenny but over 17% had never cycled in Kilkenny. The main motivators for cycling were to keep active, get fit or for health reasons and to spend time outdoors followed by environmental reasons.

More than half (55%) of respondents wanted pedestrianised areas in the town centre. Outdoor public seating was also widely looked for. Almost 30% of respondents wanted to see better access for people with disabilities. The biggest changes that respondents would like to see in the town centre were more street markets, less empty shops and more art/recreational or leisure facilities and more small specialised shops. The two most sought-after design features were spaces for outside dining and provision of cycling lanes. Almost 90% of people felt that space should be reallocated in the town centre to prioritise pedestrians and cyclists and 85% felt there were sufficient open spaces in their community. An infographic (see Figure 2 above) summarising the key results was developed and shared with stakeholders and the general public.

### 3.4. Step 4: stakeholder engagement

A thematic analysis was conducted of the transcripts from semi-structured interviews with seven key stakeholders, three community workshops, and notes and memos from over 50 meetings. The findings of the focus groups were also included in the analysis. The main themes and related subthemes emerging from the inductive thematic analysis are presented in Table 2 above. Supporting quotes reflecting the meaning of each theme are also included.

### 3.5. Step 5: systems Map

**Figure 3:**
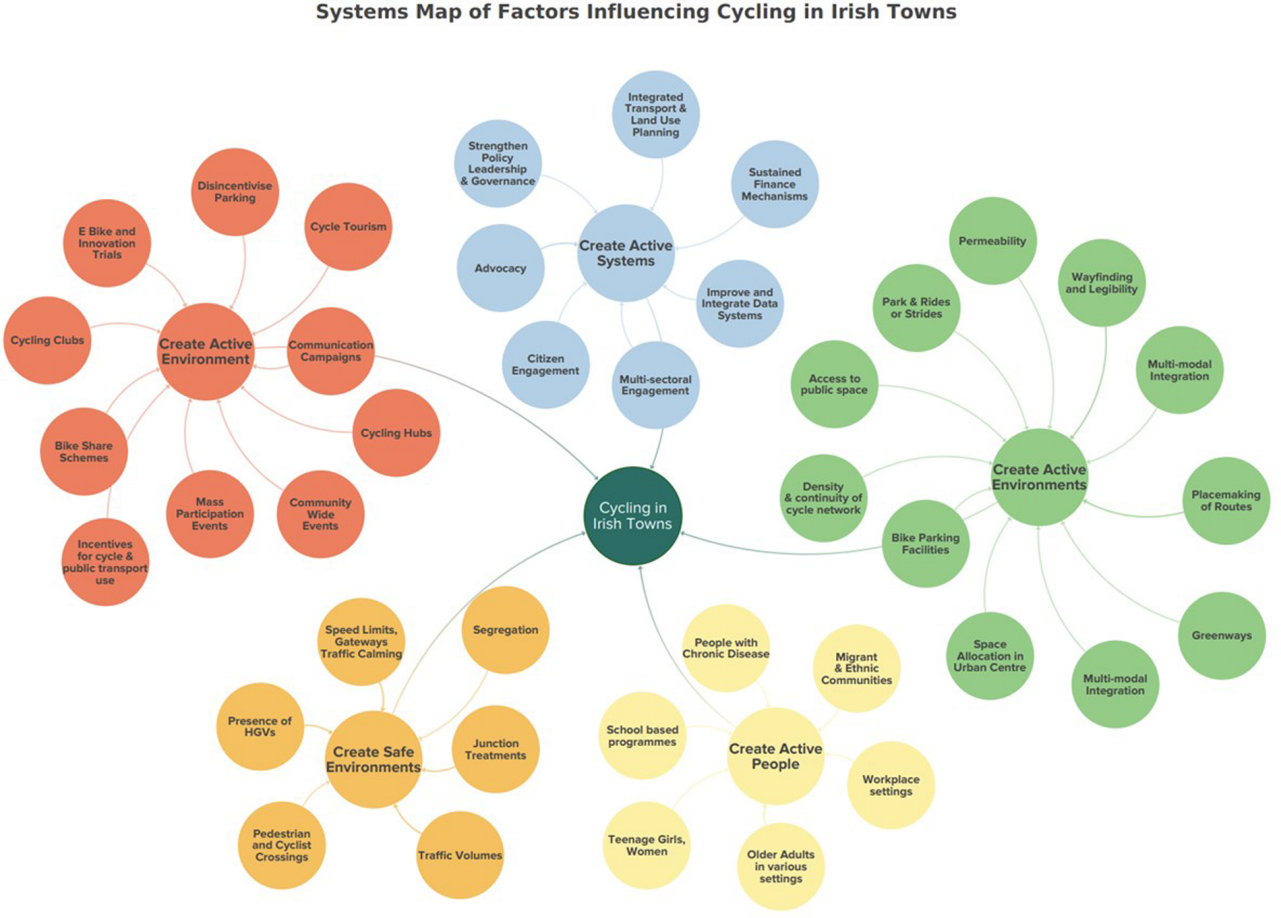
Systems Map of factors, represented as elements, influencing cycling in Irish towns.

The final systems map is presented in Figure 3 above. Causal loops will be presented as part of steps six to nine in a future paper.

## 4. Discussion

The systems approach adopted for this research allowed for a clear understanding to be gained of the process of change for large, complex systems, specific to cycling in Irish towns. This approach enabled an examination of the interrelationships in the system from multiple perspectives (57). A broad and diverse community and stakeholder engagement identified the key factors that contribute to a cycling culture and informed the development of a context specific systems map. The iteration of the systems map presented in this paper (step 5) contained five strategic objectives with all the factors or variables that influence cycling in Kilkenny depicted as elements on the map. The following section discusses the study findings for each of the broad objectives, considers the merits of using this 9-step systems approach and outlines the next steps in the process.

### 4.1. Creating active systems

The elements presented under this objective, represent the variables that underpin the system that will allow transformational change to occur. Specifically, there were seven elements which included; policy leadership and governance, integrated transport and land-use patterns, sustained finance mechanisms, multi-sectoral engagement, citizen engagement, advocacy and integrated data systems. Over the last number of decades, our transport systems have been designed in response to the growth in car ownership to facilitate increasing car use resulting in greater allocation of space in urban centres to private cars and the creation of hostile environments for pedestrians and cyclists. This has induced greater car use and car dependency and by favouring the car, urban sprawl and dispersed population patterns have ensued, resulting in reduced active modes (30). The transport system has locked in decades of investments in road infrastructure. Decades of under investment in public transport and active travel has left these modes as unattractive options accounting for small mode shares. There is consensus across stakeholders that the policy framework is now in place. The integration of transport and land use continues to be challenging. In spite of the 10 min city and in-fill policies, population forecasts predict a continuation of traditional, dispersed, settlement patterns. In Kilkenny, although the percentage of forecasted population growth is slightly higher in Kilkenny city, the absolute forecasted growth is just 4,965 compared to 15,268 outside of the two largest urban areas. Remote working may exacerbate this problem, with greater possibilities of residential self-selection. Since 2021, the programme for government has reallocated transport funding to public transport, active travel modes and somewhat less to behaviour change. The focus must move towards scaling up and increasing the pace of implementation and infrastructure roll out. Transformational, systemic change requires multi-sectoral and multi-policy action (58). The establishment of a platform of decision makers with the remit to act on all leverage points in the system is essential to bring about the necessary change. A joined-up approach allows for greater awareness of transport, land use and health challenges in urban centres, across the populations (59, 60). Community members expressed a sense of exclusion from decision making and a desire to contribute to place based solutions, participatory planning and co-creation. On the other hand, a disconnect exists between the work that has been undertaken in the city to date and the awareness of this in the community. There is a need for clear pathways for communication and feedback from the community to decision makers and vice versa. Collaborative work allows for greater understanding of the roles of others and adds value to infrastructure projects by ensuring it recognises and responds to the needs of the communities. Furthermore, an essential part of any systems approach in public health is the improvement and integration of data systems. Indicators must be identified and measurement systems adopted for tracking progress on policies, strategies and interventions (61, 62).

### 4.2. Creating active and safe environments

Under these two objectives, seventeen elements were identified and the perception of lack of safety was to the fore across all engagements. This is consistent with narratives from other car-dependent countries. Without the provision of safe, segregated cycling infrastructure, cycling is only for “the strong and the fearless”, and perhaps “the enthused and confident” (63). The design and development of a strategic cycle network featured prominently together with a reallocation of road space and reduction in traffic volumes and speeds. There are challenges with progressing from the planning to delivery phases for infrastructure projects, investments can face political obstacles and lengthy processes and take many years to deliver (64). Hence, the rapid deployment of cycling infrastructure requires reallocation of road space. Yet, this requires extensive local engagement, placing additional demands on small teams in smaller cities. Extension of car-free centres, one-way systems, filtered permeability, removal of on-street parking and reduction in carriageways is contentious and requires political support. Brave decisions on space reallocations and land use are required. More acceptable, but also long-term solutions include the provision of park and strides/rides, multi-modal hubs, attractive public spaces, green spaces and greenways. Safe, secure bike parking, light segregation, improved crossings and permeability, reductions in speed limits and traffic calming elements can be shorter term solutions and easy wins. This multi-faceted approach to redesigning the built environment is in accordance with other studies (65–67). Additionally, a strong sense of place emerged throughout the engagement process with references to the medieval city, arts and crafts, a strong sporting tradition and the built and natural heritage. There is a desire to weave this into the design of public spaces and mobility networks through placemaking. This is consistent with the findings of the Te Ara Mua Future Streets project (68), that suggests an emphasis on incorporating local traditions into mobility systems can enhance the sense of place and identity. In Kilkenny, this identity extends to the rural hinterland as does the desire for accessible connections to the city. Similarly, Nilsson (69), has suggested that placemaking is an important element in the development of a cycling culture.

### 4.3. Creating active societies and people

These two strategic objectives contain elements that relate to cultivating a cycling culture in the wider society and under-served population groups and settings. The private car has traditionally been the first choice in Kilkenny with high car ownership and modal share. The lack of alternative mobility solutions was cited frequently in the community engagement and the visibility of other transport options is poor. Cycling is regarded as a crucial element of a multi-faceted solution that views cycling not as a standalone transport mode but as a key piece in the transport offering that is highly dependent on the allocation of space and integration with public transport, park and pedal, micro-mobility and other last mile solutions in smaller cities and towns (16). Leverage points proposed throughout the engagement and also supported in the literature include the provision and availability of bike share schemes and e-bikes, incentives for cycling and public transport use (70, 71) and disincentivising car parking (72, 73). Community-wide programmes such as car-free day, Bikeweek, communication campaigns and mass participation events were also suggested and have a strong evidence base (74, 75). Other propositions included support of cycling initiatives such as cycle tourism, the development of cycle hubs and the support of clubs. Arguably, the leverage points with the greatest potential for modal shift such as disincentivising car driving are often the least feasible to implement.

The elements under Creating Active People were derived from a strong viewpoint that vulnerable road users were not catered for in a car orientated city, resulting in a loss of independent journeys and a disparity in access to social, health, education and employment opportunities. These inequalities associated with access to transport have been reported elsewhere in Ireland (76). To overcome these transport related inequalities in access to services, the system mapping process identified a targeted approach to cycle training and education programmes. This targeted approach for population groups included; people with disabilities and chronic disease, migrant and ethnic communities, teenage girls and women and older adults. To offer localised mobility solutions for the designated target groups and the wider community, the need for workplace and school based programmes were identified. Community wide cycling programmes that deliberately prioritise the needs of vulnerable road users in such a pronounced way are uncommon in the published literature (77, 78).

### 4.4. Reflecting on the process

Although, it was not a specific objective of the process, the five steps that culminated in the development of a draft systems maps helped create a vision for a cycling city in Kilkenny. The vision is a city that integrates a strategic cycle network with multi-modal provision whilst retaining the rich culture and heritage of the medieval city. The riparian, green spaces provide opportunities for accessible, connective corridors in the city, encouraging active engagement with nature. This vision, if it comes to fruition, will result in a healthier, more liveable city with greater quality of life for all citizens.

The use of a participatory systems approach in this research, allowed for an enhanced engagement process and a detailed mapping of all the variables that influence cycling in Kilkenny and in similar small cities. Subsequently, the mapped variables informed the process of identifying leverage points and involving a broad group of stakeholders in solution generation. In this way, complex problems can be disaggregated, assigned to and analysed by the stakeholders with the expertise, experiences and potential to intervene. The systems approach has resulted in the foundations for an overarching vision, engaged stakeholders and a reservoir of resources to establish a platform to bring about transformational change in the mobility system. A limitation of the systems approach is the greater investment required in the planning stages. Broad stakeholder engagement necessitates additional co-ordination and results in longer preparatory phases. However, the ensuing actions are embedded in local organisations, and are systemic and transformational.

The next steps of this research will focus on utilising this platform to operationalise the systems map. Casual loops will be identified in the systems map. Stocks and accumulations in the system such as car/bike ownership, existing infrastructure and ongoing investment, will be analysed. Interventions will be co-designed with stakeholders and mapped onto a Theory of Change Framework.

## 5. Conclusion

The paper described how a systems map was developed in a small Irish city utilising broad stakeholder engagement to map the variables that influence cycling in the town and to identify the leverage points for transformational interventions. This work adds to the development of systems approaches to tackle complex problems as it synthesises the scientific evidence, a participatory systems approach and the pragmatic findings of the embedded researcher working alongside an active travel team. The systems map will form the basis of a framework for cultivating a cycling culture in small cities and will take it beyond a theoretical framework to an organic, live process.

## Data Availability

The raw data supporting the conclusions of this article will be made available by the authors, without undue reservation.
